# Pregnancy and Crohn’s disease: concerns and assurance of medical therapy

**DOI:** 10.1093/gastro/goac055

**Published:** 2022-10-10

**Authors:** Reezwana Chowdhury, Sunanda V Kane

**Affiliations:** Division of Gastroenterology and Hepatology, Department of Medicine, Johns Hopkins University, Baltimore, MD, USA; Division of Gastroenterology and Hepatology, Department of Medicine, Mayo Clinic, Rochester, MN, USA

**Keywords:** Crohn’s disease, pregnancy, teratogenicity, inflammatory bowel disease, lactation, biologics, anti-TNF

## Abstract

Approximately 50% of patients with inflammatory bowel disease including both Crohn’s disease and ulcerative colitis are female with many being diagnosed and treated during their reproductive years. It is important for women to be in remission prior to and during pregnancy. There have been many advances in the treatment of inflammatory bowel disease, including new therapies. In this review, we summarize the currently approved medications for Crohn’s disease and their safety in pregnancy and postpartum. The totality of evidence suggests that the majority of therapies are low-risk before and during pregnancy, and should be continued to control maternal disease.

## Introduction

Approximately 3 million people are affected by inflammatory bowel disease (IBD) which consists of Crohn’s disease (CD) and ulcerative colitis (UC) [1]. The peak incidence occurs among patients in their prime reproductive years and women comprise ∼50% of IBD patients. Therefore, preconception counseling, medication management, and optimization during and after pregnancy are vital to maternal and fetal outcomes. Multiple studies and registries have shown that both maternal and fetal outcomes are improved when women are in remission prior to and during pregnancy [2]. It is well known that disease remission prior to and during pregnancy are associated with similar rates of spontaneous abortion, still birth, or congenital abnormalities to the general population [3]. Disease relapse during pregnancy increases the risk of spontaneous abortion, preterm birth, low birthweight, and small gestational age fetus [4]. Given the importance of remission prior to conception and during pregnancy, it is of utmost importance for patients to understand the need to continue therapy given the abundance of data regarding the safety of the various medical options. Below we summarize the various categories of medications including biologics, antibiotics, immunosuppressive, and some of the newer agents.

## Antibiotics

The most frequent antibiotics used in IBD are metronidazole and ciprofloxacin. Antibiotics may be indicated for pouchitis, complicated CD such as perianal or intra-abdominal abscesses, and *Clostridioides difficile*. Metronidazole is generally considered to be low-risk during pregnancy. There has been a large cohort study that has not shown any increased risk of congenital abnormalities (CA), preterm birth, or low birthweight with exposure to metronidazole [5]. Metronidazole has been detected at low levels in breastfed infants with no adverse effects from exposure [6].While metronidazole is considered low-risk, there are usually alternative antibiotics with similar coverage and better tolerance. Ciprofloxacin, a fluoroquinolone antibiotic, is associated with bone and cartilage abnormalities. There is a theoretical risk of fetal musculoskeletal development particularly during the first trimester. However, based on human studies and meta-analyses, there have not been additional CA reported; authors recommended weighing the risks vs benefits of therapy and administration under expert guidance [6, 7]. In addition, ciprofloxacin can be detected at low levels in breast milk; it is recommended to withhold breastfeeding for 4–6 hours after administration [7, 8].

## Corticosteroids

Corticosteroids are often used for flares or uncontrolled disease as a bridge and should not be used for maintenance therapy. In rheumatological data, steroids during pregnancy have been low-risk [9]. Prednisolone and prednisone are more effectively metabolized by the placenta than dexamethasone or betamethasone and fetal levels are lower than maternal levels [10]. Multiple studies have shown an increased risk of maternal and fetal complications with corticosteroid use during pregnancy, including gestational diabetes, preterm birth, low birthweight, and neonatal adrenal suppression; however, disease state during corticosteroid use may have confounded the data. The Pregnancy in Inflammatory Bowel Disease and Neonatal Outcomes registry (PIANO) did show an increased risk of gestational diabetes and low birthweight independently of disease activity [11]. In another study, however, there was no increased risk in CA, but there was a trend toward increased risk of preterm birth and infant infection within 4 months after exposure. There have also been reports of increased risk of cleft lip and palate secondary to intrapartum steroid use especially during the first trimester of pregnancy, although a recent large study with >2,300 cleft cases did not show any overall increased risk [12]. Using the prospective PIANO registry, Odufalu *et al*. [13] found that *in utero* corticosteroid use was associated with increased risk of low birthweight, preterm birth, and intrauterine growth retardation, and increased risk of infection when corticosteroid was used during the second and third trimesters. Budesonide has a high first-pass metabolism and low bioavailability that may result in less fetal exposure. There are limited data regarding its safety in pregnancy. Beaulieu *et al*. [14] evaluated 539 female patients with CD with 60 pregnancies in 41 women and found no significant increase in poor pregnancy outcome or CA, suggesting that budesonide may be safer than standard prednisone. Regarding breastfeeding, excretion of corticosteroids into breast milk is dose-dependent. Therefore, women taking >20 mg/day of prednisone should consider delaying breastfeeding for 4 hours after administration, whereas prednisolone, reaching lower serum and breast milk levels than prednisone, may be preferable when a higher dose of corticosteroids is required [15, 16].

## Immunosuppressants

### Immunomodulators

Azathioprine and mercaptopurine are among the common immunomodulators used as a steroid-sparing agent in the treatment of IBD. Both azathioprine and mercaptopurine can traverse the placenta. Fetal liver, however, is unable to metabolize azathioprine (which is the prodrug) to mercaptopurine due to lack of the enzyme iosinate pyrophosphylate reducing potential toxicity. This enzyme converts azathioprine to its active metabolites of 6-mercaptopurine and S-methyl-4-nitro-5-thioimidzaole [17]. Human studies have thus far been reassuring regarding the safety of these medications during pregnancy in IBD patients. In a French study of 215 pregnancies in 204 women with IBD, there was no difference in live births, congenital anomalies, or prematurity between patients exposed to thiopurines and those not exposed to thiopurines [18]. In another study of 187 patients treated with thiopurines, 66 patients receiving antitumor necrosis factor (anti-TNF) agents and 318 unexposed did not find any difference in pregnancy complications among the various groups, and multivariate analysis showed that treatment with thiopurine was associated with decreased rate of spontaneous abortion and no obstetric complications [19]. In the PIANO registry, there were >335 thiopurine-exposed pregnancies with no increased risk of CA or pregnancy complications, and exposed children achieved equal developmental milestones compared with those not exposed [19, 20]. It is recommended to continue thiopurine monotherapy during pregnancy to maintain remission as risks of flare outweigh risks of therapy. Thiopurines, however, generally should not be initiated during pregnancy due to slow onset of action and risk of pancreatitis [15].

Methotrexate (MTX), a folic acid analog, is used as a steroid-sparing or steroid-resistant medication in the treatment of CD. MTX irreversibly inhibits dihydrofolate reductase enzyme, which is necessary for purine synthesis, inhibiting DNA replication. MTX is a known abortifacient, showing increased risk of spontaneous abortions and CA. Women of childbearing age should be counseled about the risk of teratogenicity prior to taking MTX and educated about contraception use. Women who wish to become pregnant should stop the medication and start high-dose folic acid at least 3 months prior to attempting conception [6, 21]. In addition, this medication is not recommended during lactation.

### Anti-TNFα agents

We know that TNFα plays a role in the inflammatory pathway for both intestinal and extra intestinal inflammation in IBD. In addition, TNFα is also secreted by the placenta and helps support pregnancy [22]. Infliximab and adalimumab are IgG1 antibodies that target the neonatal Fc receptor [23, 24]. The IgG1 subclass is transported across the placenta more readily than the other subclasses and increases in the third trimester [23]. The concentrations of IgG1 subclass (infliximab and adalimumab) in the cord blood correlated inversely with timing of drug administration and the concentration of infliximab was higher than that of adalimumab in the cord blood [23, 25]. Certolizumab, another anti-TNFα agent in the armamentarium for the treatment of CD, lacks the Fc component of IgG1. It is a recombinant human Fab’ fragment bound to a polyethylene glycol molecule. Given its lack of the Fc component, certolizumab only undergoes passive transport across the placenta, unlike infliximab and adalimumab, which undergo active transport, and therefore has much lower neonatal levels than maternal levels [26]. Of the anti-TNF agents, the majority of pregnancy safety data include infliximab and adalimumab [27]. The myriad of studies including prospective registries and meta-analysis did not show any increase in worse pregnancy outcomes, increased risk of CA, preterm birth, spontaneous abort, or low birthweight [28, 29]. However, a retrospective database study by Luu *et al*. [30] showed a higher risk of maternal infections, overall maternal infections, and preterm delivery, but no fetal complications or CA. Another study by Chaparro *et al*. [31] showed an increased risk of maternal infections at 4.1% vs 0.9% and more newborn complications at 24.5% vs 16% (exposed to anti-TNFα agents vs unexposed) but no increased risk of CA, preterm delivery, or overall pregnancy complications. Given that both infliximab and adalimumab are actively transported across the placenta and there appear to be higher infliximab and adalimumab levels in neonatal cord blood than in maternal serum at birth, there is concern about the effect on child development and vaccination [25]. The mean time for adalimumab clearance in infants is 4 months while that for infliximab is 7.3 months; half-life is estimated to be 26 days for adalimumab and 33 days for infliximab [25]. In a study following children up to 47 months, there was no increased risk of malignancy or serious infection in children exposed to anti-TNF [31]. In the EVASION study, which was a retrospective study between 2011 and 2015 evaluating anti-TNF use during pregnancy, there were 11,275 pregnancies in ∼8,000 pregnancies [30]. About 1,475 women were exposed to anti-TNF with 17% having CD. There was no significantly increased risk of infection in the first year of life even with use of anti-TNF in the third trimester. There was, however, an increased risk of infection in mothers exposed to anti-TNF, similar to that in non-pregnant women; risk of infection did not increase when medication was continued beyond 24 weeks, but discontinuation before Week 24 increased risk of flare [30, 32].

Another meta-analysis did not show any increased risk of child infections, antibiotic use, or hospitalization in infants exposed to anti-TNF *in utero* [33]. The current recommendations are to avoid live vaccinations for 6–12 months in infants who were exposed to anti-TNF *in utero* [15]. However, a study by Park *et al*. [34] has shown that it is safe to administer BCG vaccination at 6 months in 90 infants exposed to anti-TNF *in utero* with last dose administered at 30-week gestation. In endemic areas with tuberculosis, it is worth discussing the risk/benefit when deciding on the administration of this vaccine. The BCG vaccine is not a recommended vaccine in the USA.

Combination therapy with thiopurine and anti-TNF is commonly used to induce and maintain remission in IBD. Thus far, the data have been reassuring for both thiopurine and anti-TNF monotherapy, and recently the PIANO registry has confirmed those findings [2]. This prospective, observational study across the USA conducted between 2007 and 2019 assessed pregnant women with IBD. There were 1,490 completed pregnancies, with 1,431 live births and 1-year infant outcomes were available for 1,010. There were 227 that were exposed to both thiopurines and biologics, 242 exposed to thiopurines, and 642 exposed to biologics; drug exposure to mono or combination therapy did not show any increased adverse maternal or fetal abnormalities [2].

Based on the assuring safety data on use of biologics, thiopurines, and combination therapy, it is key to emphasize the importance of drug continuation. Discontinuation of anti-TNF therapy in remission has a risk of relapse of ∼50% within 2 years in the general population [35, 36]. We know that premature drug cessation carries increased risk of adverse pregnancy outcomes due to flares. Most recently, in a systematic review and meta-analysis that included 48 studies with 6,963 patients, biologic therapy, biologic therapy including anti-TNF, anti-integrins, and anti-cytokines, the rate of early pregnancy loss, preterm birth, low birthweight, and congenital malformation of pregnant IBD women were comparable to those of the general population [37]. This study again showed that continuing therapy throughout the third trimester was not associated with increased risk of adverse pregnancy outcomes compared with discontinuing biologic therapy [37].

### Anti-integrin agents

Vedolizumab is a humanized monoclonal IgG1 antibody that binds to the α4β7 integrin receptor on the T-lymphocytes antagonist used for induction and maintenance in CD. The MAdCAM-1 receptor is primarily located in the gastrointestinal lymphoid tissue. This IgG1 antibody is likely to increase toward later stages of pregnancy much like the anti-TNFs. In the PIANO registry, 41 patients were exposed to vedolizumab and did not show any increased risk of pregnancy or fetal complications [2].

Animal studies have not shown any evidence of adverse effects on pre- or post-natal developmental milestones of vedolizumab. The majority of the studies on vedolizumab have been retrospective and lacking control groups [38]. The pan-European CONCEIVE retrospective study collected data on infection rates and malignancies following vedolizumab-exposed infants ≤1 year of age [39]. The multicenter study of 79 patients conducted by Moens and colleagues showed comparable rates of prematurity, CA, miscarriage, gestation age, and birthweight between the patients exposed to anti-TNF agents with and without a combination immunomodulator. The rate of serious infections did not differ between the vedolizumab-exposed cohort, the anti-TNF-exposed cohort, and the control cohort, and there were no malignancies among live-born infants. The various studies have reported seven CA including neural tube defect, hip dysplasia, pulmonary valve stenosis, Hirschsprung’s disease, and corpus callosum agenesis [6]. In addition, the level of vedolizumab detected in newborns (50%) was lower than maternal levels [40]. Vedolizumab has not been detected in infants >15 weeks post-partum [41]. According to guidelines, if vedolizumab is required during pregnancy, the last dose should be given 6–10 weeks prior to delivery and then resumed 48 hours after delivery if uncomplicated [42]. A prospective registry called the Mothers’ and Babies’ Outcomes (DUMBO) registry being conducted in 70 centers in Spain was created to assess the long-term safety of intrauterine exposure to biological and immunomodulatory agents ≤4 years of age and data collection is ongoing [43].

Another member of the integrin family is natalizumab, which is a recombinant IgG4 antibody that binds to the α4 subunit of the α4β7 subunit on circulating lymphocytes, which prevents its interaction with MAdCAM-1 in the gut and also binds to the α4 of the α4β1, which inhibits interaction with the vascular cell adhesion molecule that is also expressed on cerebrovascular cells. This medication has been used to treat multiple sclerosis and has also increased risk of progressive multifocal leukoencephalopathy [44]. This medication is non-specific to gut lymphocytes as with vedolizumab and is used less frequently in the USA in comparison to other countries [45].

Like vedolizumab, natalizumab also undergoes active transport across the placenta. The majority of human data stem from studies on women with multiple sclerosis with reports of self-limiting anemia and thrombocytopenia after exposure during the third trimester [46, 47], while other studies have not reported any increased rate of adverse outcomes [48]. Therefore, like vedolizumab, natalizumab is considered to be safe in pregnancy, and the final dose should be given 4–6 weeks prior to delivery to reduce infant drug levels [42].

### Anti-interleukin 12 and 23

Ustekinumab (Stelara^®^), a human IgG1 kappa antibody against interleukin-12/23p40, is approved for the treatment of plaque psoriasis, psoriatic arthritis, UC, and CD. Interleukin-12 and interleukin-23 are involved in the development and maintenance of pregnancy. Interleukin-12 is involved in uterine angiogenesis with abnormal levels associated with impaired embryo implantation and spontaneous abortion [49, 50]. Interleukin-23 regulates human decidual immune cells and its increased expression is associated with spontaneous abortion; like other IgG1 molecules, it gets transported across placenta during the final weeks of pregnancy. In animal studies, ustekinumab has not shown any adverse effects on fetal or neonatal development [51]. Data in human studies have also been reassuring despite its transfer via the placenta, although much of the data were based on observational studies, case reports, and post marketing surveillance [2, 52]. A study by Mahadevan and colleagues summarized data from Janssen’s Global Safety Database on ustekinumab-exposed pregnancies [53]. This analysis showed that the dose of ustekinumab, 45 or 90 mg, did not correlate with adverse pregnancy outcomes in both IBD and non-IBD indications. In addition, this study suggested that paternal exposure is not a risk factor for adverse pregnancy outcomes. The authors observed that the overall birth rate was prospectively higher in pregnancies associated with maternal exposure to ustekinumab compared to the general population (81% vs 62.9%). Rates of adverse pregnancy outcomes including live births with major CA, spontaneous abortions, and elective/induced abortions were consistent with prevalence rates in the general population in the USA. The outcome rates were similar to those reported in patients exposed to infliximab [54]. The authors of this descriptive study concluded that maternal and paternal exposure to ustekinumab immediately before or during pregnancy did not lead to increased rates of adverse pregnancy outcomes, regardless of disease indication or duration of exposure [53]. In a prospective study of 19 patients with CD by Flanagan *et al*. [52], the authors found that ustekinumab drug levels appear to be stable during pregnancy and the maximal time to clearance was 19 weeks with median time of 9 weeks, which reinforces the current recommendation to avoid live vaccination before 12 months of age.

Risankizumab (Skyrizi) is the newest interleukin inhibitor recently approved by the Food and Drug Administration (FDA) for the treatment of moderate to severe CD. It is a humanized IgG1 monoclonal antibody that selectively binds to the p19 subunit of interleukin (IL)-23. This drug had already been approved for active psoriatic arthritis and severe plaque psoriasis. The phase 3 induction studies ADVANCE and MOTIVATE were randomized, double-blind, 12-week, placebo-controlled evaluations of 600 and 1,200 mg risankizumab intravenous injection (IV) every 4 weeks. Both biologic failure and biologic naive patients were included in the studies [55]. Data thus far have shown that IV risankizumab was well tolerated and resulted in statistically significant and clinical improvements vs placebo for moderately to severely active CD with no difference in dosing between 600 and 1,200 mg and no additional safety concerns. The current recommended FDA dosing is 600 mg IV 0, 4 and 8 weeks followed by 360 mg subcutaneous at Week 12 and then every 8 weeks thereafter. Based on the data from our Rheumatology colleagues, risankizumab has already been used in plaque psoriasis; it is assumed that risankizumab does cross the placenta, but there are no significant human data available and use in pregnant women has not been studied. Based on animal studies, there is a possibility of fetal losses and neonatal deaths in monkeys [56]. At the time of this publication, with the recent approval for risankizumab, there are not enough data to support the use of risankizumab in pregnant women.

### Lactation

It is well known that there are many benefits of breastfeeding. A common concern among IBD patients on various medications for treatment is excretion of medication in breast milk. The majority of medications to treat IBD are compatible with breastfeeding including 5-aminosalicylic acid (5-ASA), steroids, immunomodulators, and biologics. While pharmacokinetics demonstrates the highest concentrations of thiopurine were during the first 4 hours of maternal ingestion, it is no longer recommended that mothers pump and dump their milk [42]. Further guidance regarding lactation is shown in Tables 1 and 2.

**Table 1.** Current medications in the treatment of Crohn's disease

Medication	Maintenance dosing	Lactation recommendations
Aminosalicylates^a^	Continue, Sulfasalazine—add 2 mg folate	Compatible, monitor infant for diarrhea—mesalamine
Sulfasalazine		
Corticosteroids	Reserve for flares	Compatible with breastfeeding; recommend nursing 4 hours post dose; limited data for budesonide, prednisolone may be preferred as lower concentrations compared to prednisone in breast milk [16]
Thiopurine	Continue as monotherapy, therapeutic drug monitoring as dosing may be altered due increased renal clearance in pregnancy	Compatible
Methotrexate	Contraindicated	Not advised
Thalidomide		
Antibiotics	Reserve for perianal disease and pouchitis, amoxicillin preferred over ciprofloxacin and metronidazole	Amoxicillin compatible
		If Metronidazole required, can be administered with close monitoring [6]

a Aminosalicylates are not effective in the treatment of Crohn’s disease and therefore not indicated in treatment.

**Table 2.** Biologics used in the treatment of Crohn’s disease

Medication	Dosing recommendation	Breastfeeding
Biologics (majority)	Maintain throughout pregnancy	Compatible
Infliximab (anti-TNF)	Base dosing on pregnancy weight during pregnancy	Compatible
Adalimumab (anti-TNF)	Final injection 2–3 weeks before delivery	Compatible
Certolizumab pegol (anti-TNF)	No dose adjustment	Compatible
Vedolizumab (anti-α4β7)	Final 6–10 weeks before delivery; if every 4 weeks, 4–5 weeks before delivery	Compatible
Ustekinumab (IL-12/23 inhibitor)	Final 6–10 weeks before delivery; if every 4 weeks, 4–5 weeks before delivery	Compatible
Risankizumab (IL-23 inhibitor)	Not enough data to recommend continuation	Not enough data, not recommended
Tofacitinib^a^ (Janus kinase inhibitor)	Not enough data to recommend continuation	Not enough data, not recommended
Upadacitinib^a^ (Janus kinase 1 inhibitor)	Not enough data to recommend continuation	Not enough data, not recommended

a Currently approved for use in ulcerative colitis.

TNF, tumor necrosis factor; IL, interleukin.

### Conclusions

The current data on medications such as antibiotics, corticosteroids, 5-ASAs, and immunosuppressive and anti-TNF agents are thus far reassuring for patients and providers. There are large databases throughout the world following women and men exposed to these medications at the time of conception and their exposed offspring. There is still concern among pregnant women and their physicians regarding continuation of these medications throughout pregnancy and lactation. The data thus far on the newer biologic agents such as ustekinumab and vedolizumab are also reassuring. The most important point for patients and physicians to understand is that women are and remain in remission at the time of conception, so preconception counseling is absolutely necessary for favorable outcomes. Women who are not in remission at the time of conception tend to have complications and women who stop medications such as biologics risk relapse, which is also associated with poorer pregnancy outcomes. As the armamentarium of medications continues to increase with newer drugs on the horizon for IBD, more research is warranted regarding the safety of these drugs in pregnancy and postpartum; however, with the wide variety that we have at this time, patients and physicians should have assurance about treatment options.

## Authors’ Contributions

R.C. and S.K. are equal contributors to the article.

## Conflict of Interest

R.C.: none. S.K.: consultant to Boehringer Ingelheim (Ridgefield CT, USA), Bristol Meyers Squibb (New York, NY, USA), Janssen Pharmaceuticals (Raritan, NJ, USA), Gilead (Foster City, CA, USA), and Takeda Pharmaceuticals (Lexington, MA, USA).
